# Dissociable Perceptual Effects of Visual Adaptation

**DOI:** 10.1371/journal.pone.0006183

**Published:** 2009-07-10

**Authors:** Kai-Markus Müller, Frieder Schillinger, David H. Do, David A. Leopold

**Affiliations:** 1 Unit on Cognitive Neurophysiology and Imaging, Laboratory of Neuropsychology, National Institute of Mental Health, Bethesda, Maryland, United States of America; 2 International Max-Planck Research School, Tübingen, Germany; 3 Department of Psychology, University of Tübingen, Tübingen, Germany; 4 Johns Hopkins University School of Medicine, Baltimore, Maryland, United States of America; University of Regensburg, Germany

## Abstract

Neurons in the visual cortex are responsive to the presentation of oriented and curved line segments, which are thought to act as primitives for the visual processing of shapes and objects. Prolonged adaptation to such stimuli gives rise to two related perceptual effects: a slow change in the appearance of the adapting stimulus (perceptual drift), and the distortion of subsequently presented test stimuli (adaptational aftereffects). Here we used a psychophysical nulling technique to dissociate and quantify these two classical observations in order to examine their underlying mechanisms and their relationship to one another. In agreement with previous work, we found that during adaptation horizontal and vertical straight lines serve as attractors for perceived orientation and curvature. However, the rate of perceptual drift for different stimuli was not predictive of the corresponding aftereffect magnitudes, indicating that the two perceptual effects are governed by distinct neural processes. Finally, the rate of perceptual drift for curved line segments did not depend on the spatial scale of the stimulus, suggesting that its mechanisms lie outside strictly retinotopic processing stages. These findings provide new evidence that the visual system relies on statistically salient intrinsic reference stimuli for the processing of visual patterns, and point to perceptual drift as an experimental window for studying the mechanisms of visual perception.

## Introduction

Following the prolonged inspection of a visual stimulus, a subsequently viewed stimulus sometimes appears conspicuously distorted. This phenomenon, termed an *adaptational aftereffect* can affect the perception of various stimulus attributes, and is an important psychophysical tool for probing how the brain encodes stimuli at many levels [1]–[3]. For instance, adapting to a tilted line causes a subsequently presented vertical line to appear tilted in the direction opposite of the adaptation line, an observation thought to tap into competing populations of orientation tuned neurons in the visual cortex [4]–[7]. Aftereffect paradigms typically involve several seconds of inspection of an adapting stimulus, followed by the brief assessment of a probe stimulus whose distortions are measured. A perceptual aftereffect therefore does not simply reflect the brain's state of adaptation, but is instead a complex interaction between the adapting stimulus, the state of adaptation, and the probe stimulus. In fact, very similar perceptual distortions often result from simultaneous, rather than sequential, presentation of the inducing (i.e. adapting) and probe stimuli (e.g. [8], for a review see [9]). These and other findings raise the question, to what extent are aftereffects determined by adaptation to the inspection stimulus, and to what extent are they a function of the relationship between the adapting and probe stimulus?

Early work by the eminent psychologist J.J. Gibson, who first described the tilt and curvature aftereffects many decades ago [10]–[12], linked aftereffects directly to the adaptation process. He first showed that, even in the absence of a probe stimulus, adaptation makes a mark on perception. Specifically, Gibson noted that during the prolonged observation of tilted lines, the line orientation would drift slowly toward the nearest cardinal (horizontal or vertical) axis. Similarly, curved line segments would subjectively become slightly straighter with time. Gibson argued that the nature of these perceptual changes revealed a framework of internal reference points, or *norms*, which served to guide visual processing. In the case of tilt, the relevant norms were the cardinal axes. In the case of curvature, the relevant norm was a straight line or edge. According to his view, adaptation involved a “recalibration”, or temporary shift, in the norm, changing one's subjective perception of what constitutes horizontal, vertical, or straight. Aftereffects, Gibson argued, were then a natural consequence of such recalibration, since probe stimuli would be interpreted in the context of a temporarily shifted norm.

Although provocative, this hypothesis of norms failed to gain traction in subsequent decades, as it was incompatible with experimental observations. For example, it was found that the tilt aftereffect could be elicited with respect to orientations other than horizontal or vertical [13], [14]. In contrast to aftereffects, which have been studied widely, only a few studies have attempted to understand the dynamic perceptual processes during prolonged inspection of stimuli [15]–[18]. Eventually, the value of adaptation and aftereffects for investigating visual processing was dismissed even by Gibson himself [19].

Nevertheless, in part based on Gibson's original conjectures, aftereffects have become the standard method for measuring visual adaptation. The present study questions the assumption that aftereffects and adaptation are inextricably linked by measuring each quantity independently. We investigate this issue using the same stimuli originally studied by Gibson: namely, tilted and curved line segments. For a set range of adapting stimuli, we compare aftereffect magnitude with the rate of perceptual drift during inspection, with the latter quantity measured using a novel dynamic nulling paradigm. We report that perception drifts toward particular stimulus values, and that the rate of this drift varies systematically with stimulus features. However, this drift rate, while scale-invariant, does not predict the magnitude of corresponding aftereffects, and therefore does not represent the same underlying process. Thus the present experiments, while supporting a norm-based account of stimulus processing, demonstrate that adaptation is, at least to some degree, distinct from perceptual aftereffects.

## Results

**Figure 1:**
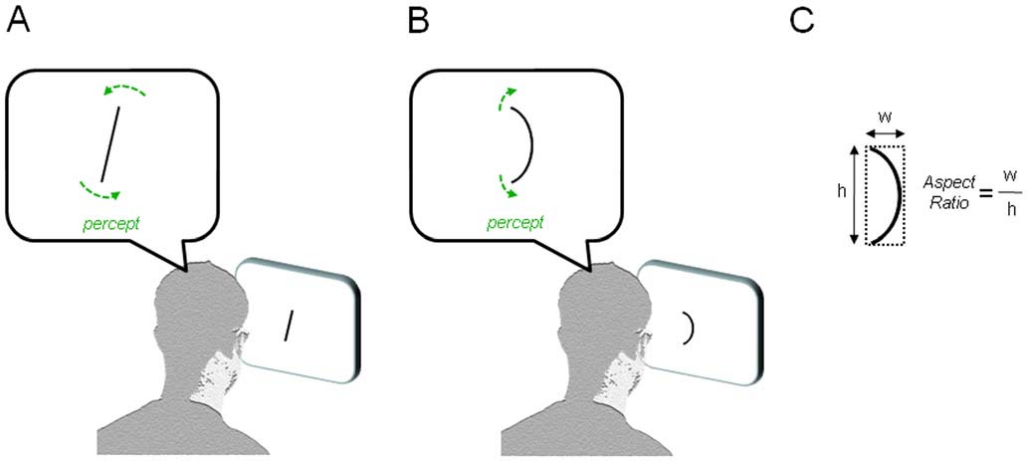
Perceptual drift during prolonged adaptation to simple, unchanging visual stimuli. A. During inspection of a line tilted slightly from vertical, the line will appear to drift toward the vertical orientation. B. During inspection of a curved line, the line will appear to straighten. C. The shape of the curves is determined through a single parameter, the unit-less aspect ratio (width/height). Stimuli are shown as black on a white background, though in the actual experiments the stimuli were white on a black background.

**Figure 2:**
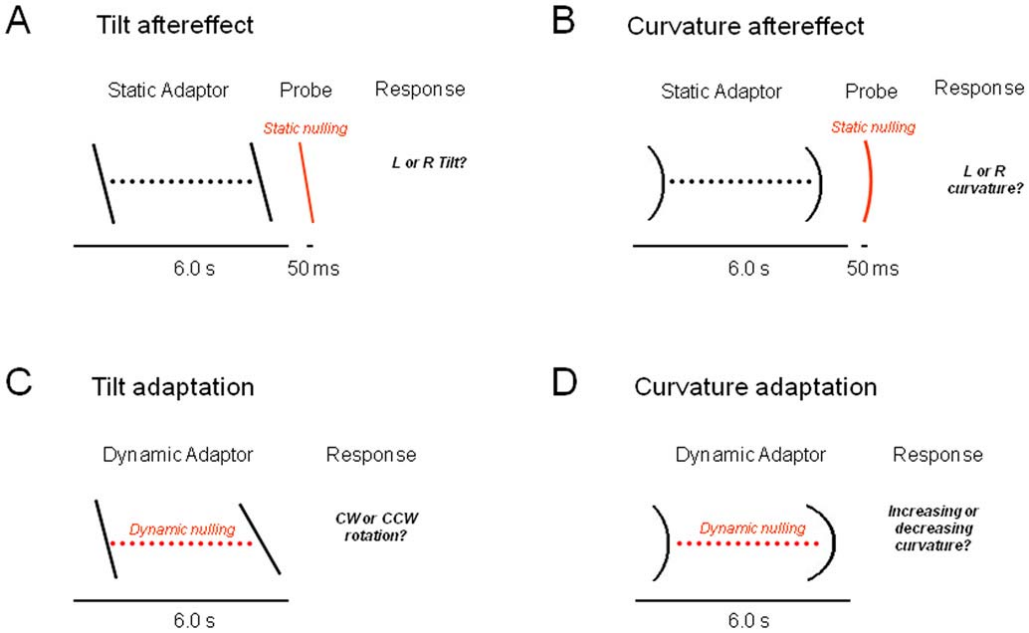
Experimental Design. The stimuli consisted of an array of 11 white dots on a black background. As subjects fixated on the center dot, a nulling procedure was implemented by means of a forced choice paradigm and an interleaved staircase procedure (see *Materials and Methods*). A. The tilt aftereffect was measured for 12 adaptation orientations evenly spaced from −90° to +90°, with the probe's nulling orientation changing by increments of .25° during the staircase. Subjects reported whether the probe stimulus was tilted clockwise or counterclockwise. B. The curvature aftereffect paradigm was similar, with the increments of .5% changes in aspect ratio (defining the curvature) during the staircase. Subjects reported whether the probe stimulus appeared more leftward or rightward. C. Changes in perceived orientation during prolonged adaptation were assessed using the dynamic nulling paradigm. The stimulus was slowly rotated during adaptation, with subjects required to report when it was stationary. Increments in the physical rate of rotation during the staircase procedure were in steps of .0625°/s. D. Dynamic nulling of the curved line was similar, with subjects asked whether the observed curve was curling more leftward or rightward. Increments in the rate of curling motion aspect ratio were .1%/s.

Our principal aims were to quantify perceptual drift upon viewing oriented and curved stimuli, to determine whether perception was drawn by specific attractors, and to study the relationship between such drift and the more commonly studied adaptational aftereffects. The main observation is depicted schematically in Figure 1. Upon several seconds of viewing lines tilted slightly away from vertical, their apparent orientation appeared to rotate very slowly, becoming slightly more vertical over time. Similarly, prolonged viewing of curved line segments resulted in their becoming slightly straighter. These observations are consistent with the notion that certain stimulus attributes – in this case verticality and straightness – play a special role in visual analysis, perhaps serving as norms for the interpretation of incoming visual patterns. Figure 2 shows the paradigm in which we applied the common psychophysical method of nulling to evaluate the strength of aftereffects for orientation and curvature. A variant of this paradigm, termed *dynamic nulling*, was then used to evaluate the rate of perceptual drift during the adaptation phase (see *Materials and Methods*).

### Orientation

**Figure 3:**
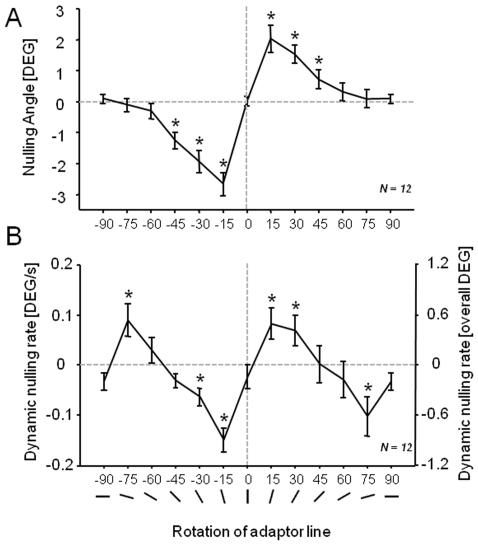
Aftereffect and adaptation with oriented line segments. A. The static nulling probe revealed that adaptation near ±15° led to the strongest aftereffect, in agreement with many previous studies. B. Adaptation to the dynamic nulling stimulus similarly revealed peaks ±15° from the vertical stimulus, as well as peaks at ±75°, corresponding to ±15° from the horizontal stimuli. Data points show average values over 12 subjects, the same individuals in A and B. Error bars represent standard error of the mean between subjects (s.e.m.). Data points marked by an asterisk (*) are significantly different from zero (2-sided t-test, p<.05, not corrected).

We first examined the effects of adapting to line segments of different orientations, both on the aftereffect magnitude in subsequently viewed probe stimuli, as well as on the perceptual drift experienced during the adaptation period itself. The results in Fig. 3A show that stimuli oriented between 15° and 45° away from vertical were effective adaptors for generating the well-studied tilt aftereffect (2-sided t-test, p<.05, not corrected). The plot, which shows the average perception of vertical across subjects, replicates many previous studies [5], [11], [20], [21]. While some studies have, in addition, reported an indirect component of tilt adaptation, i.e. an attractive shift towards the adaptor, for large adaptation angles [5], [11], [22], we did not measure any such attraction using this stimulus set and paradigm (see *Materials and Methods* for details).

We next used dynamic nulling to investigate the rate of perceptual drift during the inspection period. In these trials, we applied a small, constant rotation to the adaptor stimulus to counteract, or null, any perceived rotational drift. At the end of the 6 s adaptation period, subjects were required to indicate whether the adapting stimulus appeared to be rotating clockwise or counterclockwise, and the rate of nulling was adjusted accordingly on each trial in the staircase procedure. All orientations were interleaved during testing to avoid systematic or prolonged exposure to any orientation. Fig. 3B shows the rotation rate required to make the adapting stimulus appear stationary, as a function of orientation. We found four peak drift rates for orientations 15° away from the vertical and horizontal axes.

Thus the profile of perceptual drift differed substantially from that of adaptational aftereffects, suggesting that they represent different aspects of brain function. First the pattern of aftereffect strength showed two additional peaks as a function of adapting orientation (see Discussion). Second, the mean accumulated perceptual drift during adaptation was roughly 5× lower than the corresponding mean aftereffect size (see right axis Fig. 3B). Finally, comparing the drift rate and aftereffect size for the ±15° peaks revealed that these two factors were uncorrelated across subjects (r = −.03, p = .92).

### Curvature

**Figure 4:**
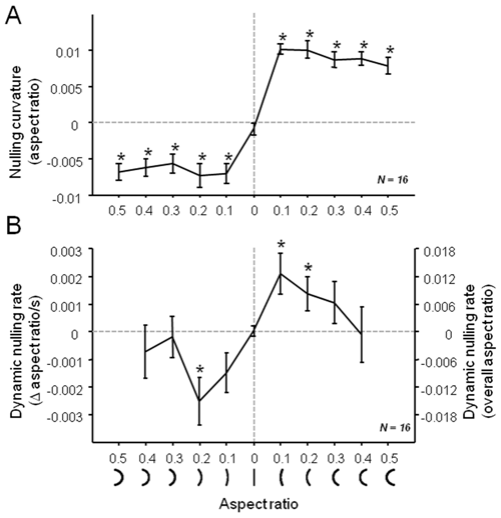
Aftereffect and adaptation with curved line segments. A. The static nulling probe revealed that aspect ratios above .1, with curvature either leftward or rightward, yielded significant curvature aftereffects. Unlike the tilt aftereffect, there was no clear peak, and no significant difference between the aftereffect strength for the 5 adaptors of equal sign. B. In contrast to the aftereffect, the dynamic nulling paradigm revealed peaks in the rate of “uncurling”, for adapting stimuli with aspect ratios between .1 and .2. Data points show average values over 16 subjects, the same individuals in A and B. Error bars represent s.e.m.. Values marked by an asterisk (*) are significantly different from zero (2-sided t-test, p<.05, not corrected).

We next repeated the experiments with upright curved line segments, using static and dynamic nulling to modulate the level of curvature in the context of a staircase. Curvature was defined as the aspect ratio of an imaginary rectangle into which the arc of a circle was inscribed (see Fig. 1B and *Materials and Methods* for details). Fig. 4A shows the aftereffect to a range of adaptor curves, measured by applying a nulling orientation to a probe stimulus, which began the staircase testing as a straight line. On each trial subjects indicated whether the line was curved leftward or rightward. Given the plotted average “points of subjective straightness”, all adaptor stimuli tested (other than the straight line segment) were effective in inducing a negative after-effect (2-sided t-test, p<.05, not corrected). There was no difference in aftereffect magnitude for the 5 adaptors of equal sign (repeated measures one-way ANOVA∶ F(4, 60) = .68, p = .61 for aspect ratios <0 and F(4, 60) = 2.21, p = .08 for aspect ratios >0).

As with orientation, the dynamic nulling of curvature revealed systematic perceptual drift, as indicated by the rate of the nulling required for the stimulus to maintain an apparently constant curvature level (Fig. 4B). During adaptation, subjects were required to indicate their perception of curling motion, which was adjusted according to the adaptive staircase procedure. The rate of subjective curling during adaptation peaked at intermediate curvature levels, and then declined at high curvature levels.

Thus, as with orientation, adaptation to curvature revealed a dissociation between the pattern of aftereffect magnitudes and perceptual drift rates. For the same adapting stimuli, aftereffect magnitudes were largely insensitive to curvature levels, while drift rates showed clear peaks. (However, it is important to point out that for stimuli with smaller absolute curvature, perceptual drift might be more readily detectable). Furthermore, as with orientation, the magnitudes of static and dynamic nulling for individual subjects were uncorrelated (for aspect ratios of ±.2∶ r = −.11, p = .68, for aspect ratios of ±.1∶ r = −.17, p = .53). For curvature, however, the maximal rate of perceptual drift did correspond well to the maximal aftereffect, as can be seen on the axis plotted on the right of Fig. 4B.

### Size Constancy

The peaks observed in the rate of perceptual drift offer two distinct explanations. The maximal rate might be determined by absolute curvature (1/radius) or by the relative curvature (shape). Absolute curvature can be thought of as the local orientation gradient present on the retina, whereas shape is scale-invariant, and defined in the present experiments by the unit-less aspect ratio of the imaginary rectangle into which the curves are inscribed. By this definition, changing the scale of the adaptor stimulus, while holding the aspect ratio constant, would affect the absolute but not relative curvature. Some theoretical and neurophysiological work suggests that the brain's encoding of geometric curvature might be related to neural activity in strictly retinotopic areas of the brain with small receptive fields, such as V1 or V2 [23]–[25]. If adaptive processes in these areas were to underlie the observed effects, one might expect absolute curvature to be the relevant stimulus parameter. On the other hand, the observed size constancy for shape responses in other parts of the brain, such as the inferotemporal cortex [26], [27], suggests that there could also be adaptive processes in which the relative curvature, or ”shape”, was the important feature.

**Figure 5:**
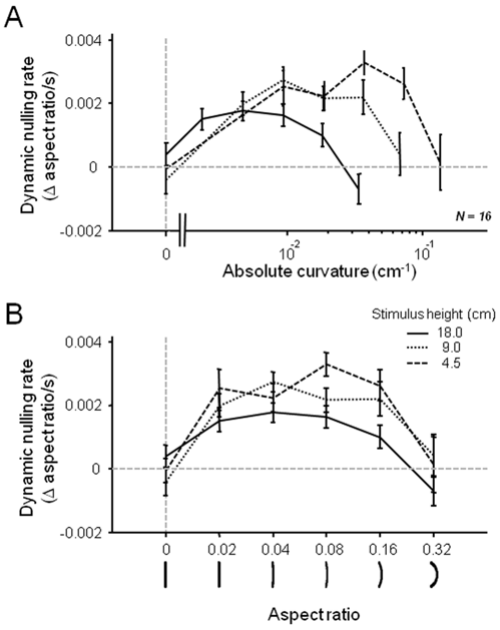
Size constancy of perceptual drift for curvature. Adaptors of three sizes ranging over a factor of 4 were used to determine effect strength of adaptation through dynamic nulling (N = 16 subjects, partially overlapping those in Figure 4). The display was 114 cm, i.e. 1 cm foveally corresponds to ½ deg visual angle. Since that approximation is invalid for larger values, units are given in cm. A. Data plotted against absolute curvature, with smallest stimuli having the highest curvature (dashed line) and largest stimuli having the lowest curvature (solid line). B. *The exact same data* plotted against aspect ratio for the three different sizes. Note that aspect ratio, rather than absolute curvature, determined the rate at which the stimulus perceptually straightened. Data were collected for convexities in both directions and then combined. Error bars represent s.e.m over subjects.

To distinguish these possibilities, we repeated the dynamic nulling experiments for curvature, varying the size of the adaptor stimuli. The largest stimuli were inscribed into an imaginary rectangle that was 4 times taller (and 16 times larger in area) than the smallest stimuli. This variation resulted in the same aspect ratio having different values of absolute curvature (see *Materials and Methods* section and Table 1 for details). The adaptor stimuli were spaced logarithmically, to a range of 0.32, with both directions of curvature presented. Fig. 5 shows the data for the three different sizes, with the leftward and rightward adaptor curvature combined. Fig. 5A shows the data plotted in terms of absolute curvature, in units of 1/radius (cm^−1^). A repeated measures 2-way ANOVA revealed, for the three levels of absolute curvature in which the stimuli overlapped (.009, .018, .035), a significant interaction between the factors *size* and *curvature* (F(4,60) = 5.63, p<.001). This was not the case when the same data were aligned in terms of their aspect ratio (Fig. 5B). While the size played some role in the overall rate of adaptation, the curves had the same basic shape and did not show any interaction between *size* and *aspect ratio* (repeated measures 2-way ANOVA (over .02, .04, .08, .16, .32), F(8,120) = .97, p = .46). These results, taken together, indicate that the aspect ratio and thus the “gestalt”, rather than the absolute curvature, was the primary determinant for the rate of adaptation.

**Table 1:** Parameters of stimuli used in the size constancy experiment.

Large stimuli				
**Width(cm)**	**Height(cm)**	**Aspect Ratio**	**Radius(cm)**	**Curvature**
0.00	18.00	0.00	∞	0.000
0.36	18.00	0.02	450.18	0.002
0.72	18.00	0.04	225.36	0.004
1.44	18.00	0.08	113.22	0.009
2.88	18.00	0.16	57.69	0.017
5.76	18.00	0.32	31.01	0.032
Medium stimuli				
**Width(cm)**	**Height(cm)**	**Aspect Ratio**	**Radius(cm)**	**Curvature**
0.00	9.00	0.00	∞	0.000
0.18	9.00	0.02	225.09	0.004
0.36	9.00	0.04	112.68	0.009
0.72	9.00	0.08	56.61	0.018
1.44	9.00	0.16	28.85	0.035
2.88	9.00	0.32	15.50	0.065
Small stimuli				
**Width(cm)**	**Height(cm)**	**Aspect Ratio**	**Radius(cm)**	**Curvature**
0.00	4.50	0.00	∞	0.000
0.09	4.50	0.02	112.55	0.009
0.18	4.50	0.04	56.34	0.018
0.36	4.50	0.08	28.31	0.035
0.72	4.50	0.16	14.42	0.069
1.44	4.50	0.32	7.75	0.129

When doubling aspect ratio of the imaginary box the curve is inscribed in, curvature does not automatically double. The current stimulus set represents an optimized combination of width and height of the stimuli in order to be able to compare curvatures and overall shape.

## Discussion

Visual features and objects are normally perceived to be stable in their appearance, with any observed changes assumed to reflect changes in the world rather than adjustments in the brain of the percipient. Such perceptual stability is remarkable, given the ubiquity of adaptive processes affecting visual analysis, from the retina to the association cortex [28]–[31]. The present experiments investigate the exception to the rule, and measure the relatively minor penetration of adaptation into our perceptual experience. We show that these changes, though subtle, are quantifiable and consistent.

### 

#### Two faces of visual adaptation

It is arguable that adaptational aftereffects, which are an important part of the psychologist's toolbox, do not provide a pure assessment of the adaptive process itself, since it involves two stimuli. While increased adaptation time strengthens aftereffects, the interaction between adapting and test stimuli may not be fundamentally grounded in dynamic, adaptive processes. For example, repulsion in perceived orientation associated with the simultaneous tilt *illusion* (also reported by Gibson [12] closely resembles the tilt aftereffect in many respects, but does not entail adaptation [8], [32]. Furthermore, very brief presentations of an adaptor stimulus can in some cases lead to subsequent misperceptions in shape [33], [34] or orientation [35]. These repulsive effects are presumably based on the interaction between stimuli rather than on adaptation processes [9].

The dynamic nulling paradigm used here isolates the adaptation process without invoking a second probe stimulus, thereby avoiding the potential confounds of stimulus-stimulus interaction. Our results suggest that adaptational aftereffects and perceptual drift derive, at least to some extent, from distinct neural mechanisms. First, with both the tilted and curved stimuli, there was no correlation among subjects between the rate of perceptual drift and the magnitude of the corresponding aftereffect. This is despite a large overall variation in drift rates and aftereffect sizes across subjects. Second, for the oriented lines (but not the curves), the accumulated orientation drift over the adaptation period was insufficient to explain the tilt aftereffect magnitude, falling short by a factor of five.

Finally, for both oriented and curved lines, the pattern of aftereffect magnitudes differed from that of drift rates as a function of the adapting stimulus. This effect is clear in the case of curvature, where intermediate and high curvature adaptors elicited similar aftereffects, but gave rise to very different rates of perceptual drift. For orientation, the nature of the mismatch between Fig. 3A and Fig. 3B requires more explanation. The aftereffect and the drift share peaks at ±15° from vertical, suggesting that they may be related. However, previous experiments have shown that the position of these peaks depends on the reference stimulus, appearing at ±15° on either side of *any* axis chosen as a reference orientation, not only the cardinal axes (see also Figure S1 and [13], [14], [36], [37])). In contrast, the four orientation peaks found in the Fig. 3B involved no such choice of a reference, and therefore represent genuine perceptual attractors. What might the basis be for the observed differences between perceptual drift and the subsequent aftereffects? An obvious conclusion is that they draw upon at least partially distinct underlying neural mechanisms.

Aftereffects are inherently spatial and, with few exceptions [34], [38], are only effective when the adapting and test stimuli are presented to the same portion of the retina. Accordingly, most models of the well-studied tilt aftereffect invoke elements that are characteristic of neural responses in retinotopic areas such as V1 [4], [6], [7]. For many stimuli, the nature of the figural distortion can be reduced to a spatial repulsion of portions of the test stimulus from portions of the adapting stimulus, and are observed similarly even in the absence of adaptation, when the “adapting” and test stimulus are presented simultaneously (e.g. the tilt illusion [8], [9]). In contrast, perceptual drift involves only one stimulus, whose changing appearance depends on the brain's response to it, but not on its interaction with a second stimulus. Both perceptual drift and aftereffects depend on adaptation time. For example, the strength of the tilt aftereffect grows logarithmically with adaptation duration, saturating only after an hour of exposure to the adapting stimulus [39]. It would be of great interest to know how changes in the rate of perceptual drift over time relate to the dynamics of aftereffect buildup.

What determines the direction of perceptual drift? Our results show that observed changes are governed by non-retinotopic, higher-level stimulus attributes. For example, it was not the *absolute* curvature level that determined the rate of perceptual drift (Figure 5), but rather then shape itself (i.e. the aspect ratio of the box into which the curve was inscribed). For orientation, other work found that perceptual drift of tilted lines follows a world-centered (as opposed to retinotopic) frame of reference [15]. A previous hypothesis suggested that such perceptual changes reflected the gradual decay of initial *overestimation* of stimulus properties such as orientation and curvature [17]. In all cases, the consistent direction of perceptual drift underscores the brain's apparent tendency to consider some stimuli to be special reference points, or norms. This heuristic dates back to Gibson, who invoked the concept of norms to account for both drift and aftereffects. Recently, this idea has been applied to applied to a wide range of higher-level stimulus attributes, including even the identity of faces [3], [40]–[42], whose aftereffects also show a logarithmic buildup over time [43]. The present study shows for two categories of stimuli that the perceptual attraction to such norms during periods of adaptation can be measured and quantified, and ultimately disentangled from the spatial interactions associated with aftereffects.

### Tuning the brain to the world

The processing of orientation and curvature in the brain's retinotopic areas is based on two dimensional projections of three-dimensional objects and scenes. As frequently pointed out, images on the retina are locally ambiguous with respect to the third dimension and continually require inferences about the structure of the external world. Our eye and brain are specialized for this task, and able to extract visual information in a highly efficient manner [44], with adaptive processes thought to remove inefficient redundancy in the coding [45] (for reviews, see [46], [47]). The brain's adaptation to the natural world may hold the key to understanding misperceptions of orientation, as well as our correct perceptions. It has been argued that the tilt illusion ultimately has its roots in the statistical relationship between objects in natural visual scenes, and their oriented projections on the retina [48], as does the tendency to perceive vertical line segments longer than horizontal ones of the same length [49]. Overall, horizontal and vertical orientations have a much higher prevalence in natural scenes than do other orientations [50], [51], perhaps owing to the reliable orientations of the horizon and gravity, respectively. This factor seems to have shaped responses in the primary visual cortex, as there is an over representation of cardinal orientations in the primary visual cortex of ferrets [52] and primates [53], [54], including humans [55].

In some ways, our results speak against perceptual drift arising from strictly low-level, retinotopic visual processing. It is possible that norms for seemingly low-level attributes, such as orientation and curvature, could also emerge at later stages of the visual hierarchy. More likely, such specializations for particular stimulus types are coordinated between multiple stages of visual processing. The brain's specialization for cardinal axes may also explain other psychophysical phenomena related to orientation, such as the so-called *oblique effect*, where patterns oriented along the horizontal or vertical axes are more readily detected and discriminated than those oriented obliquely [56]–[58]. Less is known about the environmental statistics of curvature, whose representation in the brain is a topic of active investigation [59]–[61]. Psychophysically, the brain appears to treat curved and straight line elements as categorically different entities, since small amount of curvature are easily detectable among otherwise straight elements, even if their orientations are randomized [62].

In summary, our sensation and perception of objects in the world involves the extensive analysis of orientation of curvature. As we inspect a stimulus, we have the impression that our perception is unbiased and stable in its analysis. However, these and other results show that, even for these primitive scene elements, our sampling of the world is biased toward certain stimuli, and continually readjusts itself, even though these changes nearly always escape our notice.

## Materials and Methods

### Subjects

The Institutional Review Board of the National Institutes of Health approved all procedures and written informed consent was obtained for all subjects. Twelve subjects participated in the orientation experiment reported in Figure 3 (age range: 20–38, mean 26.4 years, 5 female, 7 male). 8 were naïve to the hypothesis of the experiment. All subjects had normal or corrected to normal vision. For different conditions within one experiment (orientation, curvature, or size constancy respectively) the same subjects were used allowing us to compare individual effect sizes of adaptation and after-effect. The order of the experiments was counterbalanced across subjects. Sixteen subjects participated in the curvature experiment reported in Figure 4 (age range: 22–34, mean 26.0 years, 8 female, 8 male), 14 of which were naive. In the size constancy experiment reported in Figure 5, 16 subjects participated (age range: 22–38, mean 27.3 years, 6 female, 10 male), among them 11 naïve according to the hypothesis. Subjects gave written consent and were compensated for their participation.

### Apparatus and Stimuli

Subjects sat in a dark room with their chin placed on a chin rest to ensure constant eye to screen distance. For orientation and size constancy (Figures 3 and 5), the distance to the screen was set to 114 cm and for curvature (Figure 4) the distance was set to 50 cm. For the display of the stimulus sequence a Notebook screen was used (Sony VAJO, 37×23 cm, 1920×1200 pixels). The visual patterns and the stimulation sequence were designed by means of custom written code (OpenGL and C++ in Microsoft Visual Studio.NET, Microsoft, Redmond WA). Adaptation and test stimuli were white (for orientation and size constancy: 310 cd/m^2^; for curvature: 75 cd/m^2^;) presented on a black background (all Experiments: .6 cd/m^2^). The lines were created using an array of 11 white circular, evenly spaced dots, each extending .05° visual angle for orientation and curvature. Because the size constancy experiment used scaled versions of the stimulus, the dot size was .025°, .05°, or .1° visual angle respectively. Curved lines were constructed by inscribing a curve into an imaginary rectangle, with an aspect ratio of 0 relating to a straight vertical line and an aspect ratio of .5 relating to a half circle. All tested curves were constructed from vertically upright rectangles. Here, we define the degree of curvature as the aspect ratio (width/height) of that imaginary rectangle. The dotted lines generated for the orientation experiment extended 5.0° visual angle. In the curvature experiment, the height of the lines was 5.1° visual angle and the width varied using the following values: 0°, ±.52°, ±1.03°, ±1.55°, ±2.05° corresponding to aspect ratios of 0, .1, .2, .3, or .4 respectively. In the size constancy experiment a screen distance of 114 cm was used, i.e. 1 cm foveally corresponds to ½° visual angle. Curvature is defined as 1/r. Table 1 gives the values for the different stimulus parameters.

### Control measures

During each experiment, subjects were given a brief rest each 5 minutes, during which time they were required to fill out a questionnaire indicating state of alertness and fixation quality of the previous run. This mainly served as a reminder and control of crucial aspects of the task. After each experiment was completed, the subject was asked to fill out a final questionnaire asking for the hypothesis of the experiment. None of the naïve subjects guessed the hypothesis of the drift experiments correctly.

### Experimental paradigms

In the aftereffect experiments, a trial consisted of the following sequence, outlined in Figures 2a and 2b. A fixation dot appeared on the screen, which subjects were instructed to fixate throughout the trial. After 1.0 s, ten additional dots appeared, forming the adaptor stimulus, either a line segment or a curve. The original fixation dot served as an integral part of the adaptor and probe stimuli. The adaptation stimulus was presented for 6.0 s, followed by a 50 ms test stimulus after a 200 ms gap. Following each trial, the subjects responded in a forced-choice manner via mouse click whether the test stimulus appeared to be oriented clockwise or counterclockwise of vertical (tilt aftereffect) or curved with the ends of the line pointing leftward or rightward (curvature aftereffect). The point of subjective equality was attained by adjusting the test stimulus orientation or curvature (“static nulling”) according to a staircase procedure [63]. Staircases corresponding to all twelve orientations or all eleven curvatures were randomly interleaved; with each staircase terminating once 6 reversals were reached. The experiment was finished, as soon as all staircases had terminated.

In the perceptual drift experiments, outlined in Figures 2c and 2d, subject also fixated for 1.0 s and then adapted for 6.0 s. However, during these experiments, there was no probe stimulus, and nulling occurred during adaptation itself. A tone at the beginning of the intertrial interval indicated to the subject to respond whether the adaptor stimulus was rotating clockwise or counterclockwise (in the tilt experiments) or becoming more or less curved (in the curvature experiments). In a staircase design similar to the aftereffect experiments, real rotation and or curvature motion was added to the adaptor stimulus in order to bring their percept of any motion to zero (“dynamic nulling”). This was achieved by changing the width of the imaginary rectangle into which the curve was inscribed. We chose the width, because the drift illusion for curvature has been shown to be width-dependent [17]. As above, the staircases were randomly interleaved, and terminated individually once 6 reversals were reached. In order to maximally spend any residual aftereffects, a straight test line segment was added for 2.0 s between curvature trials (not depicted in Figure 2D). In the final experiment comparing different stimulus sizes, this intervening straight stimulus was not used, and the adaptation stimulus was only shown for 3.0 s.

## Supporting Information

Figure S1 (0.07 MB PDF)

## References

[1] Frisby JP (1979). Seeing: Illusion, brain and mind.

[2] Barlow HB, Földiák P, Miall C, Durbin RM, Mitchinson GJ (1989). Adaptation and decorrelation in the cortex.. The Computing Neuron.

[3] Clifford CWG, Rhodes G (2005). Fitting the mind to the world - Adaptation and after-effects in high-level vision.

[4] Coltheart M (1971). Visual feature-analyzers and after-effects of tilt and curvature.. Psychol Rev.

[5] Clifford CW, Wenderoth P, Spehar B (2000). A functional angle on some after-effects in cortical vision.. Proc Biol Sci.

[6] Jin DZ, Dragoi V, Sur M, Seung HS (2005). Tilt aftereffect and adaptation-induced changes in orientation tuning in visual cortex.. J Neurophysiol.

[7] Liu T, Larsson J, Carrasco M (2007). Feature-based attention modulates orientation-selective responses in human visual cortex.. Neuron.

[8] Wenderoth P, van der Zwan R (1989). The effects of exposure duration and surrounding frames on direct and indirect tilt aftereffects and illusions.. Percept Psychophys.

[9] Schwartz O, Hsu A, Dayan P (2007). Space and time in visual context.. Nat Rev Neurosci.

[10] Gibson JJ (1933). Adaptation, after-effect and contrast in the perception of curved lines.. J Exp Psychol.

[11] Gibson JJ, Radner M (1937). Adaptation, after-effect, and contrast in the perception of tilted lines.. J Exp Psychol.

[12] Gibson JJ (1937). Adaptation, after-effect, and contrast in the perception of tilted lines. II. Simultaneous contrast and the areal restriction of the after-effect.. J Exp Psychol.

[13] Köhler W, Wallach H (1944). Figural After-Effects: An Investigation of Visual Processes.. Proceedings of the American Philosophical Society.

[14] Mitchell DE, Muir DW (1976). Does the tilt after-effect occur in the oblique meridian?. Vision Res.

[15] Prentice WCB, Beardslee D (1950). Visual ‘normalization’ near the vertical and horizontal.. J Exp Psychol.

[16] Held R (1963). Localized normalization of tilted lines.. Am J Psychol.

[17] Coren S, Festinger L (1967). An alternative view of the “Gibson normalization effect”..

[18] Templeton WB (1972). Visual tilt normalization: The method of kinesthetic matching.. Percept Psychophys.

[19] Gibson JJ (1979). The ecological approach to visual perception.

[20] Wolfe JM (1984). Short test flashes produce large tilt aftereffects.. Vision Res.

[21] Wolfe JM, O'Connell KM (1986). Fatigue and structural change: two consequences of visual pattern adaptation.. Invest Ophthalmol Vis Sci.

[22] Morant RB, Harris J (1965). Two different after-effects of exposure to visual tilts.. Am J Psychol.

[23] Dobbins A, Zucker SW, Cynader MS (1987). Endstopped neurons in the visual cortex as a substrate for calculating curvature.. Nature.

[24] Dobbins A, Zucker SW, Cynader MS (1989). Endstopping and curvature.. Vision Res.

[25] Hegde J, Van E (2000). Selectivity for complex shapes in primate visual area V2.. J Neurosci.

[26] Logothetis NK, Sheinberg DL (1996). Visual object recognition.. Annu Rev Neurosci.

[27] Ito M, Tamura H, Fujita I, Tanaka K (1995). Size and position invariance of neuronal responses in monkey inferotemporal cortex.. J Neurophysiol.

[28] Enroth-Cugell C, Shapley RM (1973). Adaptation and dynamics of cat retinal ganglion cells.. J Physiol.

[29] Maffei L, Fiorentini A, Bisti S (1973). Neural correlate of perceptual adaptation to gratings.. Science.

[30] Sanchez-Vives MV, Nowak LG, McCormick DA (2000). Membrane mechanisms underlying contrast adaptation in cat area 17 in vivo.. J Neurosci.

[31] Miller EK, Gochin PM, Gross CG (1991). Habituation-like decrease in the responses of neurons in inferior temporal cortex of the macaque.. Vis Neurosci.

[32] Wenderoth P, Smith S (1999). Neural substrates of the tilt illusion.. Aust N Z J Ophthalmol.

[33] Suzuki S (2001). Attention-dependent brief adaptation to contour orientation: a high-level aftereffect for convexity?. Vision Res.

[34] Suzuki S, Cavanagh P (1998). A shape-contrast effect for briefly presented stimuli.. J Exp Psychol Hum Percept Perform.

[35] Sekuler R, Littlejohn J (1974). Letter: Tilt aftereffect following very brief exposures.. Vision Res.

[36] Taylor MM, Parnes RA (1972). Mapping the tilt after-effect..

[37] Campbell FW, Maffei L (1971). The tilt after-effect: a fresh look.. Vision Res.

[38] Hartmann L, Ellis WD (1923). Further studies of gamma movement.. A source book of gestalt psychology.

[39] Greenlee MW, Magnussen S (1987). Saturation of the tilt aftereffect.. Vision Res.

[40] Leopold DA, O'Toole AJ, Vetter T, Blanz V (2001). Prototype-referenced shape encoding revealed by high-level aftereffects.. Nat Neurosci.

[41] Rhodes G, Jeffery L (2006). Adaptive norm-based coding of facial identity.. Vision Res.

[42] Jiang F, Blanz V, O'Toole AJ (2006). Probing the visual representation of faces with adaptation: A view from the other side of the mean.. Psychol Sci.

[43] Leopold DA, Rhodes G, Müller K-M, Jeffrey L (2005). The dynamics of visual adaptation to faces.. Proc R Soc Lond B Biol Sci.

[44] Burgess AE, Wagner RF, Jennings RJ, Barlow HB (1981). Efficiency of human visual signal discrimination.. Science.

[45] Sharpee TO, Sugihara H, Kurgansky AV, Rebrik SP, Stryker MP (2006). Adaptive filtering enhances information transmission in visual cortex.. Nature.

[46] Clifford CW, Webster MA, Stanley GB, Stocker AA, Kohn A (2007). Visual adaptation: neural, psychological and computational aspects.. Vision Res.

[47] Webster MA, Werner JS, Field DJ, Clifford CW, Rhodes G (2005). Adaptation and the phenomenology of perception.. Fitting the mind to the world: adaptation and after-effects in high-level vision.

[48] Howe CQ, Purves D (2005). Natural-scene geometry predicts the perception of angles and line orientation.. Proc Natl Acad Sci U S A.

[49] Howe CQ, Purves D (2002). Range image statistics can explain the anomalous perception of length.. Proc Natl Acad Sci U S A.

[50] Switkes E, Mayer MJ, Sloan JA (1978). Spatial frequency analysis of the visual environment: anisotropy and the carpentered environment hypothesis.. Vision Res.

[51] Coppola DM, Purves HR, McCoy AN, Purves D (1998). The distribution of oriented contours in the real world.. Proc Natl Acad Sci U S A.

[52] Coppola DM, White LE, Fitzpatrick D, Purves D (1998). Unequal representation of cardinal and oblique contours in ferret visual cortex.. Proc Natl Acad Sci U S A.

[53] Mansfield RJ (1974). Neural basis of orientation perception in primate vision.. Science.

[54] Xu X, Anderson TJ, Casagrande VA (2007). How do functional maps in primary visual cortex vary with eccentricity?. J Comp Neurol.

[55] Furmanski CS, Engel SA (2000). An oblique effect in human primary visual cortex.. Nat Neurosci.

[56] Appelle S (1972). Perception and discrimination as a function of stimulus orientation: the “oblique effect” in man and animals.. Psychol Bull.

[57] Meng X, Qian N (2005). The oblique effect depends on perceived, rather than physical, orientation and direction.. Vision Res.

[58] Westheimer G, Beard BL (1998). Orientation dependency for foveal line stimuli: detection and intensity discrimination, resolution, orientation discrimination and vernier acuity.. Vision Res.

[59] Pasupathy A, Connor CE (1999). Responses to contour features in macaque area V4.. J Neurophysiol.

[60] Hegde J, Van E (2007). A comparative study of shape representation in macaque visual areas v2 and v4.. Cereb Cortex.

[61] Muller KM, Wilke M, Leopold DA (2009). Visual adaptation to convexity in macaque area V4.. Neuroscience.

[62] Fahle M (1991). Parallel perception of vernier offsets, curvature, and chevrons in humans.. Vision Res.

[63] Cornsweet TN (1962). The staircase-method in psychophysics.. Am J Psychol.

