# Supplementary material for: Dissociable Perceptual Effects of Visual Adaptation
Source: PLoS One. 2009 Jul 10;4(7):e6183. doi: 10.1371/journal.pone.0006183 (PMC2703777; doi:10.1371/journal.pone.0006183)
Supplement: Figure S1 — (0.07 MB PDF) [file pone.0006183.s001.pdf]

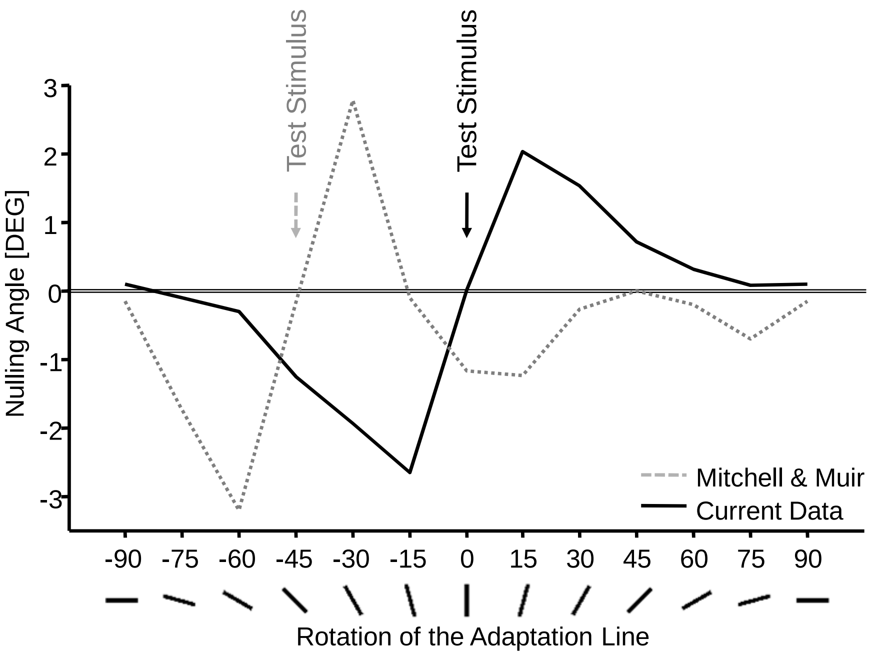

Supplementary Figure 1

Supplementary Figure 1. Dependence of after-effects on the choice of test stimuli. Data from a publication of Mitchell & Muir (1976) have been replotted for comparison with the current data. Mitchell & Muir had 3 experienced psychophysical observers judge the after-effect in the oblique meridian using a matching task of a test pattern to a line in unadapted space . The three individual data sets from Mitchell & Muir were manually read out, reversed in polarity to match the current procedure and if necessary interpolated to match our adaptation orientations. Note the high similarity of curves despite largely different procedures. This comparison shows that the tilt after-effect maxima are strongly dominated by the test stimulus chosen. A second-order commonality between these two data sets and the perceptual drift data set in Figure 3B is, that given the current spacing of adaptation orientations, positive and negative extremes are always 30° apart.
